# Red blood cell distribution width in pregnancy: a systematic review

**DOI:** 10.11613/BM.2018.030502

**Published:** 2018-10-15

**Authors:** Panagiotis Paliogiannis, Angelo Zinellu, Arduino A. Mangoni, Giampiero Capobianco, Salvatore Dessole, Pier Luigi Cherchi, Ciriaco Carru

**Affiliations:** 1 Department of Biomedical Sciences, University of Sassari, Sassari, Italy; 2 Department of Clinical Pharmacology, College of Medicine and Public Health, Flinders University, Adelaide, Australia; 3 Department of Clinical, Surgical and Experimental Sciences, University of Sassari, Sassari, Italy

**Keywords:** anisocytosis, RDW, pregnancy, preeclampsia, gestational diabetes

## Abstract

Anisocytosis has been associated with the severity and prognosis of several acute and chronic diseases, as well as physiological conditions such as pregnancy. Anisocytosis is quantified by the red blood cell distribution width (RDW), expressed as the ratio, multiplied by 100, between the standard deviation (SD) of red blood cell volumes and the mean corpuscular volume, or as the SD of erythrocyte volumes (RDW-SD). The aim of the present review was to report the state of the art on the physiological values and the putative diagnostic and prognostic roles of RDW in complicated pregnancy. Literature research for articles published in the last ten years was conducted in Pubmed, Web of Science, ClinicalTrials.gov, and Scopus databases. Abstracts were independently screened by two investigators. If relevant, full articles were retrieved. References, in these articles, citing relevant reviews or original studies were also accessed to identify additional eligible studies. Any disagreement between the reviewers was resolved by a third investigator. A total of 28 studies were included in the review. These studies reported changes in RDW values during physiological pregnancy, and associations between the RDW and several pregnancy complications including anaemia, preeclampsia, gestational diabetes, and recurrent miscarriage. This review provides background information for establishing physiological and pathological RDW values in pregnancy for diagnostic and prognostic use in clinical practice.

## Introduction

Anisocytosis, the alteration of the normal volume of red blood cells (RBC), is characterized by a high intrinsic plasticity of the external membrane and low haemoglobin content, which allow a certain degree of expansion or contraction in response to physiological or pathological stimuli (*1*). Anisocytosis is quantified by the red blood cell distribution width (RDW), expressed as the ratio between the standard deviation (SD) of RBC volumes and the mean corpuscular volume (MCV), multiplied by 100 (RDW-CV), or as the SD of erythrocyte volumes (RDW-SD). Red blood cell distribution width is particularly useful in the differential diagnosis of anaemias and other pathological conditions that lead to anisocytosis. Higher RDW values reflect the presence of anisocytosis, that may be attributable to the presence of small and large RBCs or both, while values below the lower limit of the reference interval are rare and clinically meaningless (*2*).

Recently, several inexpensive and widely available haematological indexes including the neutrophil to lymphocyte ratio (NLR), monocyte to lymphocyte ratio (MLR), platelet to lymphocyte ratio (PLR), mean platelet volume (MPV), and RDW have gained scientific interest because of their ability to reflect the systemic inflammatory state of the organism and have been associated with the severity and prognosis in several acute and chronic diseases, including cancer (*3*-*7*). Furthermore, the kinetics of RDW have been recently studied in several physiological conditions, such as ageing, physical activity and pregnancy, in order to establish physiological and abnormal reference ranges (*8*, *9*).

Pregnancy is characterized by physiologic changes that might affect, either directly or indirectly, the haematological parameters. Moreover, several pregnancy complications including hypertension, gestational diabetes, acute inflammatory conditions and miscarriage can also influence these parameters. Therefore, the determination of RDW changes associated with physiological or pathological pregnancy might be useful, as an additional tool for diagnosis, prognosis, and monitoring. The aim of the present review was to describe the available scientific evidence pertaining to physiological RDW values, and associations between RDW and complications in pregnancy.

## Materials and methods

A comprehensive literature search for articles published in the last 10 years was conducted in Pubmed, Web of Science, Scopus, and ClinicalTrials.gov databases, using the following terms: “RDW AND pregnancy” OR “red cell distribution width AND pregnancy”. The PRISMA guidelines for systematic reviews and meta-analyses were followed.

Inclusion criteria for the selection of the articles were: 1) studies involving humans; 2) studies reporting RDW values in relation to physiological and pathological conditions in pregnancy; 3) studies published in the last decade in order to avoid the confounding effect of older methods for RDW determination, and 4) articles written in English. Studies with insufficient or irrelevant laboratory and clinical data and individual case reports were excluded. Case series reported in more than one article were only considered once. Abstracts were independently screened by two investigators. If relevant, full articles were retrieved. References, in these articles, citing relevant reviews or original studies were also accessed to identify additional eligible studies. Any disagreement between the reviewers was resolved by a third investigator.

Despite our literature search was limited to the last 10 years in order to avoid technical and methodological limitations of older studies, only one of the diagnostic studies included was performed with the STARD criteria. For this reason, relevant data, like confidence intervals (CI), precise P values, *etc.*, lack in some instances, and we could not provide them. This is the current literature on the topic and no methodologically better studies are available. The spirit of this review was to include all the available studies in the modern scientific literature, with their limitations, in order to illustrate the current knowledge on the topic.

## Results

**Figure 1:**
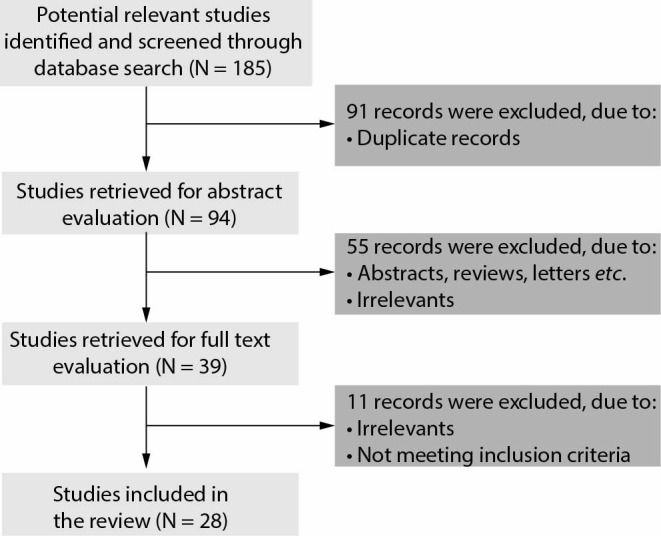
Flow chart of the search strategy followed for the selection of the articles included in the review

We initially retrieved 185 studies. Of these, 91 duplicates were excluded after an initial screening, and 55 further studies were subsequently excluded after abstract evaluation (Figure 1). After full-text review of the remaining 39 articles, eleven studies were excluded because they did not meet the inclusion criteria. Finally, 28 studies were included in the review (*10*-*37*). Among them, three articles reported RDW values in physiological pregnancy, seven articles focused on anaemia, seven on preeclampsia, and eleven articles on other complications such as cholestasis, diabetes, pancreatitis, and ectopic pregnancy.

## Discussion

### RDW in physiological pregnancy

Despite RDW has been introduced in clinical practice several decades ago, normal reference ranges in pregnancy are not well-established. This depends on both the well-known analytical variability issues related to the measurement of RDW, and the small number of large scale studies performed. Older studies evidenced contrasting RDW changes in relation to the duration of pregnancy (*38*, *39*). This uncertainty remains in more recent studies. Li *et al.* reported in a prospective study a temporal increase in RDW-CV reference intervals (2.5th and 97.5th percentile, confidence intervals, CI) in 3060 healthy pregnant Chinese women: 11.9% (11.8-11.9) - 16.8% (16.4-18.0), 12.3% (12.2-12.4) - 17.2% (16.5-18.5), and 12.3% (12.2-12.3) - 19.8% (19.3-20.5) in the first, second and third trimester, respectively (*10*). The median RDW values were significantly different between non-pregnant and pregnant women (12.3% *versus* 13.5%, respectively, P < 0.001). Khan *et al.* reported RDW-CV values higher than 15.0% in the 36.2% of the cases in a cross-sectional study including 152 healthy pregnant women in the third trimester in Pakistan (*11*). The authors suggest that the global RDW increase in the third trimester might depend on a greater bone marrow activity and/or a higher incidence of microcytic anaemia. In this study the sensitivity and specificity of RDW in detecting microcytic anaemia was 82.3% and 97.4%, respectively (*11*). Nevertheless, Amah-Tariah *et al.*, did not report any significant changes in the mean (standard deviation, SD) RDW-CV values, in a prospective study on 220 healthy Nigerian women, between the three trimesters of pregnancy (13.9% ± 0.8, 13.8% ± 0.9, and 13.9% ± 1.1, respectively, P > 0.05) (*12*). These findings highlight the need for larger prospective international studies that investigate RDW reference ranges and progressive temporal changes during pregnancy in different populations.

### RDW in complicated pregnancy

Several studies have investigated the roles of RDW in the diagnosis and monitoring of pregnancy-associated complications (Table 1).

**Table 1:** Studies investigating the role of RDW in pregnancy complications

**Authors, year, country (Ref.)**	**Design**	**Patient groups**	**Differences between cases and controls**
**ANAEMIA**			
Sultana *et al.*, 2011, Bangladesh (*13*)	P	77 non-IDA and 113 with IDA	NA
Abdelrahman *et al.*, 2012, Sudan (*14*)	P	137 non-IDA and 57 with IDA	NA
Tiwari et al. 2013, India (*15*)	P	48 with anaemia (34 IDA) and 52 non-anaemic, 2^nd^ and 3^rd^ trimester	NA
Tasneem *et al.,* 2016, Pakistan (*16*)	P	27 non-IDA women *vs*. 50 IDA	19.3% ± 10.0 *vs*. 18.6% ± 5.3, P = 0.965
Bresani Salvi *et al.*, 2016, Brazil, (*17*)	P	139 with anaemia, 2^nd^ - 3^rd^ trimester, and iron supply	NA
Schoorl *et al.*, 2012, The Netherlands (*18*)	P	25 IDA, 3^rd^ trimester, before and after iron supplements	45.0 fL ± 3.6 *vs*. 52.3 fL ± 7.0, P < 0.001
De Sà *et al.*, 2015, Brazil (*19*)	P	29 anaemic mothers and their new-borns (N = 26) in comparison to non-anaemic mothers (N = 25) and their new-borns (N = 24)	anaemic mothers: 14.3% ± 1.1 *vs*. 14.1% ± 0.8, P > 0.250 new-borns: 15.0% ± 0.8 *vs*. 15.2% ± 0.9, P = 0.870
**PREECLAMPSIA**			
Kurt *et al.*, 2013, Turkey (*20*)	P	52 PE women *vs*. 50 healthy controls	14.1% ± 1.1 *vs*. 16.9% ± 1.7, P < 0.001
Abdullahi *et al.,* 2014, Sudan (*21*)	P	65 PE women *vs*. 65 controls	14.5% ± 1.8 *vs*. 14.4% ± 1.4, P = 0.710
Gezer *et al.*, 2016, Turkey (*22*)	R	137 PE women, 150 healthy controls	14.3% ± 2.7 *vs*. 15.5% ± 2.6, P = 0.047
Reddy *et al.*, 2016, India (*23*)	P	143 (76 mild and 67 severe) PE women *vs*. 911 healthy controls	18.0% ± 3.4 *vs*. 14.8% ± 3.1 *vs*. 12.8% ± 2.1, P < 0.001
Yücel and Ustun, 2017, Turkey (*24*)	R	109 (27 mild PE and 82 severe) PE women *vs*. 110 healthy controls	13.6% *vs*. 13.8% *vs*.14.8%, P = 0.037
Yilmaz *et al.*, 2016, Turkey (*25*)	R	118 PE women *vs*. 120 healthy controls	15.2% ± 1.96 *vs*. 14.4% ± 1.70, P = 0.021
Sen-yu and Chao, 2016, China (*26*)	R	149 PE women *vs*. 70 healthy controls (20^th^ week)	13.1% ± 2.3 *vs*. 13.3% ± 2.5, P = 0.001
**GESTATIONAL DIABETES**			
Erdogan *et al.*, 2014, Turkey (*27*)	R	68 women with GD *vs*. 61 healthy controls	14.4% *vs*. 14.3%, P > 0.05
Yildiz *et al.*, 2016, Turkey (*28*)	P	53 women with GD *vs*. 35 controls	15.6% ± 1.1 *vs*. 13.0% ± 0.6, P = 0.007
Cheng *et al.*, 2016, China (*29*)	P	118 GD women with high ACR, 216 GD controls	13.6% ± 0.9 *vs*. 12.5% ± 0.6, P < 0.001
**INTRAHEPATIC CHOLESTASIS**			
Yayla Abide *et al.*, 2017, Turkey (*30*)	R	84 ICP women *vs*. 145 controls	15.1% ± 2.7 *vs*.17.1% ± 3.4, P < 0.001
Yilmaz *et al.*, 2017, Turkey (*31*)	P	90 ICP women *vs*. 90 controls	14.2% ± 1.71 *vs*. 15.2% ± 1.5, P = 0.003
**MISCARRIAGE**			
Dundar *et al.*, 2015, Turkey (*32*)	R	60 women with miscarriage history *vs*. 60 with 1^st^ trimester pregnancy *vs*. 60 parous women	16.3% ± 4.6 *vs*. 14.2% ± 3.1, P = 0.001
Yilmaz *et al.*, 2015, Turkey (*33*)	R	120 women with recurrent miscarriage *vs*. 120 controls	13.8% ± 1.4 *vs*. 14.0% ± 1.6, P =0.203
**OTHER COMPLICATIONS**			
Ilhan *et al.*, 2016, Turkey (*34*)	R	14 women with AP *vs*. 30 controls	48.9 fL ± 6.8 *vs*. 40.5 fL ± 3.0, P < 0.001
Yayla Abide *et al.*, 2017, Turkey (*35*)	P	46 women with histologically diagnosed PIA *vs*. 100 controls	15.1% ± 1.8 *vs*. 17.1% ± 3.3, P < 0.001
Beyazit *et al.*, 2017, Turkey (*36*)	P	54 women with HEG *vs*. 58 controls	12.1% ± 1.4 *vs*. 12.6% ± 1.7, P > 0.05
Akkaya *et al.*, 2017, Turkey (*37*)	R	93 women with EP and MTX therapy *vs*. 60 women with EP and surgery	12.5% ± 1.8 *vs*. 13.2% ± 1.8, P = 0.001

#### Anaemia

Anaemia is a common complication in pregnancy, affecting approximately 50% of pregnant women (*40*). It may be caused by several factors including acute infections, chronic inflammation, haemoglobinopathies and single or combined deficiency of nutrients such as folic acid, vitamin B12 and iron (iron deficiency anaemia, IDA). The latter is the predominant form and it is characterized by altered serum ferritin and transferrin concentrations and saturation, total iron binding capacity (TIBC), as well as by microcytosis (reduced red blood cell volume) and hypochromia (reduced red blood cell haemoglobin content). Therefore, RDW is one of the most useful diagnostic markers in IDA, allowing the adoption of prompt iron supplementation. Sultana *et al.* evaluated the role of RDW in the detection of iron deficiency anaemia in pregnancy within the first 20 weeks of gestation in a cross-sectional study involving 190 pregnant women in Bangladesh (*13*). Women were divided in two groups, one with IDA (serum ferritin < 12 µg/L) and one without (serum ferritin 12 to 200 µg/L); the former was further divided in latent, mild and moderate iron deficiency on the basis of transferrin saturation. The RDW showed 82.3% sensitivity, 97.4% specificity, 99.7% positive predictive value (PPV), 78.9% negative predictive value (NPV), and 88.4% accuracy for the detection of all stages of iron deficiency in pregnancy (*13*).

A similar study was performed in Sudan with the aim to compare the diagnostic accuracy of RDW and serum ferritin in IDA in 194 pregnant women with a gestational period of 21.4 ± 6.5 weeks (*14*). In this study, the sensitivity, specificity, PPV, and NPV of RDW in detecting IDA were 43.8% (95% CI: 31.4 - 57.0), 73.7% (95% CI: 65.8 - 80.5), 41.0% (95% CI: 29.2 - 53.6), and 76.0% (95% CI: 68.1 - 82.6), respectively. The authors concluded that RDW has a poor performance in diagnosing IDA among pregnant women compared with serum ferritin. Nevertheless, in an Indian study investigating correlations between blood count indexes and serum ferritin in the second and third trimester of pregnancy, it was shown a weak negative correlation between RDW-CV and serum ferritin (Pearson’s correlation coefficient, r: - 0.42, P = 0.013) (*15*). ROC analysis showed an area under the curve (AUC) of 0.77, sensitivity 82.4%, and specificity 75.8% for a RDW-CV cut-off value of 16.3% in detecting IDA. These contrasting results suggest that the diagnosis of IDA in pregnancy requires a comprehensive clinical and laboratory evaluation, in accordance with recent guidelines (*41*).

RDW has also been tested as a marker for the differential diagnosis between IDA and Beta thalassemia trait (BTT) in a cross-sectional study, however no significant differences were observed in RDW-CV values between 50 IDA and 27 BTT patients (19.3% ± 10.0 *vs*. 18.6% ± 5.3, P = 0.965) (*16*). Furthermore, RDW failed to predict responsiveness to iron supplementation therapy (*17*, *18*). De Sà *et al.* studied RDW values in new-borns of anaemic and non-anaemic women. The RDW-CV mean (SD) values in anaemic mothers were similar to that of non-anaemic mothers (14.3% ± 1.1 *vs.* 14.1% ± 0.8, P > 0.250), and no significant differences were observed between the corresponding new-borns (15.0% ± 0.8 *vs.* 15.2% ± 0.9, P = 0.870) (*19*). The authors hypothesized that iron stores in the foetus are not adversely affected by mild to moderate maternal anaemia, because of a compensatory mechanism which allows iron transport across the placenta, regardless of maternal blood concentrations.

The results of these studies suggest that RDW is often found altered in anaemic pregnants, especially those with microcytic anaemia and it can be useful in the clinical evaluation of pregnancy anemias when integrated with other haematological laboratory and clinical findings. It does not seem to have any particular effectiveness in monitoring iron supplementation therapies or predicting anaemia in new-borns; also in these cases RDW should be integrated and interpreted with other laboratory and clinical data.

#### Preeclampsia

Several studies investigated the role of RDW as a predictor of the occurrence and severity of preeclampsia. Preeclampsia (PE) is defined by new onset hypertension and proteinuria after 20 weeks of gestation in pregnancy, and represents a major cause of maternal and perinatal morbidity and mortality. The causes of PE are not clear; abnormal placenta development that results in hypoxia and ischemia is the most credited pathogenetic hypothesis, but cardiovascular maladaptation to pregnancy, several genetic, immunological and angiogenic mechanisms, as well as inflammation seem to play an important role in the pathogenesis of PE (*25*). Inflammation may produce alterations in RDW values, similar to those observed in hypertension and other cardiovascular conditions (*3*, *4*). This leaded to several studies investigating the role of RDW as a predictor of the occurrence and severity of PE (*20*-*26*).

Most of the studies performed in Asia reported correlations between RDW and the presence and severity of PE, but this finding was not confirmed in a study performed in Sudan. Kurt *et al.* reported higher mean (SD) RDW-CV values in 52 patients with preeclampsia (35 mild and 17 severe) in comparison to 50 controls (14.1% ± 1.1 *vs.* 16.9% ± 1.7, P < 0.001) (*20*). In addition, a retrospective study performed on 137 patients with preeclampsia and 150 normotensive pregnant women showed a statistically significant difference in mean (SD) RDW-CV values between the groups (14.3% ± 2.7 *vs.* 15.5% ± 2.6, P = 0.047); ROC analysis of RDW-CV at a cut-off level of 15.3% showed poor outcomes (*22*). Better results were reported by Reddy *et al*. who investigated on 143 patients with preeclampsia (76 mild and 67 severe) and 111 normal pregnancy cases. Mean (SD) RDW-CV was significantly higher in patients with severe preeclampsia in comparison to those with mild preeclampsia and normal controls (18.0% ± 3.4, 14.8% ± 3.1 and 12.8% ± 2.1, respectively; P < 0.001) (*23*). Furthermore, ROC analysis showed a good AUC (0.77, 95% CI: 0.70 - 0.83), a sensitivity of 71.3% and specificity of 65.0% (no CI provided) of RDW-CV at a cut-off level of 15.9% in discriminating mild from severe PE. Also, Yücel *et al*, Yılmaz *et al*. and Sen-yu *et al.* reported similar findings (*24*-*26*). On the contrary, Abdullahi *et al.* including 65 cases and controls matched in their basic characteristics found no difference in the mean (SD) RDW-CV between women with preeclampsia and controls (14.5% ± 1.8 *vs.* 14.4% ± 1.4, P = 0.710). In this study, there was also no difference in the mean RDW between women with mild and severe preeclampsia (14.7% ± 1.9 *vs.* 13.9% ± 1.4, P = 0.144) and, in logistic regression, there was no association between RDW and preeclampsia (OR = 0.9, 95% CI = 0.7 - 1.1, P = 0.952) (*21*). Overall, these findings suggest that RDW may be increased in preeclampsia, especially in severe cases, but such an increase remains to be better quantified, and evaluated in terms of clinical applicability, given the fragmented data reported on the sensitivity, specificity and other diagnostic accuracy parameters of RDW in the studies published so far.

#### Gestational diabetes

Gestational diabetes mellitus (GDM) is the most common metabolic disease in pregnancy, and may have deleterious effects for both the mother and the foetus. The role of RDW in predicting GDM has been evaluated recently in two studies with contrasting results (*27*, *28*). The first study was performed retrospectively in 68 patients with GDM and 61 healthy controls (*27*). Gestational diabetes mellitus was assessed by a one-hour 50-g oral glucose tolerance test (OGTT) performed between 24–28 weeks of gestation; in patients with a blood glucose concentration of more than 7.8 mmol/L, the diagnosis of GDM was confirmed by a subsequent three-hour 100-g OGTT. The values of RDW, along with other blood cell count indexes were measured and analysed, but no significant difference was detected between the median (range) RDW values in women with GDM in comparison to those without (14.4 (12.3 - 17.0)% *vs.* 14.3 (13.0 - 22.9)%, P > 0.05).

The second study, published in 2016 by Yildiz *et al.*, reported opposite results (*28*). The authors performed a cross-sectional case–control study of 53 euthyroid normotensive GDM patients and 35 healthy pregnant women. The gestational age and the definition of GDM were similar to those by Erdogan *et al.* Several blood count indexes were found to be altered in the GDM group in this study, including RDW; the mean (SD) RDW values were 15.6% ± 1.1 and 13.0% ± 0.6 (P = 0.007) in GDM and healthy controls respectively. According to the authors, the mechanism of the increase of RDW in GDM is not clear, and may be secondary to oxidative stress and rapid erythrocyte elimination, similarly to other forms of diabetes (*28*).

Cheng *et al.* investigated a different potential diagnostic use of RDW in GDM (*29*). Using a cross-sectional design, they studied 334 pregnant women with GDM in order to evaluate the role of RDW in predicting early renal injury. Depending on urine albumin, women were divided in a case (N = 118) and control group (N = 216). The authors found that the case group had a higher mean (SD) RDW-CV values (13.6% ± 0.9 *vs.* 12.5% ± 0.6, P < 0.001), that were positively associated with the albumin - creatinine ratio. Multiple logistic regression analysis showed that RDW remained independently associated with early-stage renal injury, after adjusting for several other potential cofounders. The encouraging findings of the studies performed by Yilmaz *et al.* and Cheng *et al.* suggest that RDW may be useful in the evaluation of GDM and its complications; however, further research in larger populations is warranted to establish potential clinical applications in this context.

#### Cholestasis

Abide *et al.* investigated the role of RDW in predicting the severity of intrahepatic cholestasis (IHC) in pregnancy in a retrospective case-control study including 229 pregnants, 84 with ICP and 145 age-matched healthy controls (*30*). IHC was defined as serum bile acid concentrations ≥ 10 µmol/L with pruritus that could not be explained by any other condition; the patients with ICH were further divided into two groups according to their serum bile acid concentrations: mild (< 40 µmol/L, N = 53) and severe (≥ 40 µmol/L, N = 31). The mean (SD) RDW-CV values were significantly lower in patients with IHC than in controls (15.1% ± 2.7 *vs.* 17.1% ± 3.4, P < 0.001), but no alterations in association with the severity of the disease were evidenced (*30*).

Yilmaz *et al.* published a similar prospective case-control study including 90 pregnant women with IHC and 90 controls; definitions of IHC and its severity were similar to those by Abide *et al* (*31*). In this study the mean RDW values were significantly lower in the IHC group than in the controls (14.2% ± 1.71 *vs.* 15.2% ± 1.5, P = 0.003). In contrast to the findings of Abide *et al.* however, the mean (SD) RDW-CV was significantly associated to the severity of the disease, being higher in the severe IHC patients (15.8% ± 1.1) than in those with mild disease (14.9% ± 1.6, P = 0.006). Furthermore, in correlation analyses in IHC group RDW was correlated with the total bilirubin and transaminase concentrations. The authors advocated that elevated bile acid concentrations may lead to the release of several proinflammatory mediators and trigger inflammatory response in hepatocytes which affect blood count cell indexes, such as the RDW that was found to be increased in several chronic liver diseases (*31*). This could explain the higher RDW values detected in more severe IHC in relation to milder conditions, however it does not reconcile with the lower valuesin IHC in comparison to healthy controls reported in both the studies. In any case, the diagnostic accuracy of RDW, and the ability to stratify patients with IHC, needs to be better clarified.

#### Recurrent pregnancy loss

The kinetics of RDW in women with recurrent pregnancy loss have been evaluated by Dundar *et al.* in 2014 (*32*). The authors performed a retrospective analysis of the main blood count indexes in 60 women who had a history of recurrent pregnancy loss, 60 healthy women who had a first trimester pregnancy, and 60 healthy parous women. Recurrent miscarriage was defined as three or more consecutive first-trimester (< 13 weeks) miscarriages, two or more second trimester miscarriages (13–24 weeks) or one third trimester foetal loss (> 24 weeks) combined with at least one first-trimester miscarriage, excluding cases due to uterine anomalies and/or endocrine abnormalities. Double thrombophilia tests were also performed at an interval of 12 weeks, in order to evaluate associations with the indexes under investigation. The authors found that mean (SD) RDW-CV was significantly higher in women with recurrent pregnancy losses than in pregnant women (16.3% ± 4.6 *vs.* 14.2% ± 3.1, P = 0.001), and in the 19 women with thrombophilia than in the 41 without (18.4% ± 5.8 *vs.* 15.3% ± 3.6, P = 0.043). They hypothesized that anisocytosis could increase the thrombotic predisposition of red blood cells; it is not clear whether anisocytosis is the underlying cause, or an epiphenomenon due to pre-existing conditions (*32*).

The findings of Dundar *et al.* regarding the higher RDW values in women with recurrent pregnancy loss were not confirmed in a more recent study which retrospectively included 120 women with recurrent miscarriage and 120 match-paired controls (*33*). In this study the mean (SD) RDW-CV values were somewhat lower in cases than in controls (13.8% ± 1.4 *vs.* 14.0% ± 1.6, P = 0.203), but the difference was not statistically significant. These studies did not asses the accuracy of RDW values in predicting recurrent miscarriage. In addition, their results highlight significant uncertainties regarding the kinetics of RDW in women with multiple miscarriages. Further well-designed prospective studies are necessary to evaluate the predictive or diagnostic accuracy and clinical usefulness of RDW in this setting.

#### Other pregnancy complications

A few studies have been published to date, investigating the roles of RDW variations in other rarer pregnancy complications. Ilhan *et al.* in 2015 studied several blood cell count inflammatory markers including RDW as early predictors of acute pancreatitis (AP) in pregnancy, and potential associations of these markers with AP severity (*34*). The study retrospectively involved 14 pregnant patients with AP, and 30 healthy pregnant controls; the mean (SD) RDW-SD values were significantly higher in patients with pancreatitis (48.9 fL ± 6.8 *vs.* 40.5 fL ± 3.0, P < 0.001). The study has several limitations, like the retrospective design and the small number of patients, but the results suggest a potential role of the RDW in predicting acute pancreatitis in pregnancy.

In another cross-sectional study, the role of RDW in predicting placental invasion abnormalities (PIA) was evaluated in 46 women with histologically confirmed PIA (*35*). The control group included 100 women with suspected but not histologically confirmed PIA. Mean (SD) RDW-CV values were significantly lower in women without PIA than in those with PIA (15.1% ± 1.8 *vs.* 17.1% ± 3.3, P < 0.001), and in univariate and multivariate analysis they were independently associated with PIA absence.

RDW was tested also in the evaluation of hyperemesis gravidarum (HEG), a medical condition of intractable vomiting during pregnancy affecting approximately 0.3 - 2% of all pregnancies (*36*). The study was performed in 54 HEG patients and 58 age- and gestational-age-matched controls, but no statistically significantly differences with RDW-CV were detected (12.1% ± 1.4 *vs.* 12.6% ± 1.7, P > 0.05)

One study investigated the role of RDW in the context of establishing adequate therapeutic strategies. In particular, the study retrospectively evaluated the role of several blood cell count indexes in predicting the most accurate treatment in patients with ectopic pregnancy (EP) (*37*). There were 153 EP cases, 93 treated with methotrexate (MTX) and 60 with surgery. Red blood cell distribution width was significantly increased in MTX group (13.2% ± 1.8 *versus* 12.5% ± 1.8, P = 0.001). Furthermore, RDW values were independently associated with MTX therapy in univariate and multivariate analyses. In ROC analysis, the best RDW-CV cut-off value was 13.4% (sensitivity 73.3%; specificity 40.9%), with a poor although significant AUC (0.66, 95% CI: 0.57 - 0.75, P = 0.001). The authors advocate that the RDW could be useful in selecting the right therapy in patients with EP.

## Future perspectives

Anisocytosis is involved in several phases of physiological pregnancy, as well as in several pregnancy complications. The studies available in the current scientific literature and described in this review provide encouraging evidence supporting the potential clinical involvement of RDW in predicting pregnancy complications; nevertheless, these studies offer a fragmented view of such an involvement, and are often retrospective or characterized by several biases; indeed, only one of the diagnostic studies enrolled was performed in accordance with the STARD guidelines. Moreover, it is well-known that one of the leading technical issues in routine assessment of RDW is that the reference range is highly analyser-dependent (*2*). This greatly limits the establishment of RDW reference intervals in a huge variety of physiological conditions and diseases (including pregnancy), and the availability of homogeneous data to analyse. Thus, deviations from the reference intervals documented in several pregnancy complications in the studies enrolled in this review cannot be homogeneously evaluated. Large well-designed studies performed with the same laboratory technologies and protocols are necessary to establish the exact reference ranges of RDW during the different phases of pregnancy in several populations and in women with different anthropometric, demographic, and biological characteristics. Furthermore, large prospective studies are warranted to evaluate the kinetics of RDW in women with specific pregnancy complications, possibly defined with widely accepted criteria, and again, harmonized laboratory approaches. This may be of significant utility considering that the RDW is a simple, low-cost, easy to perform, and widely available index.

In conclusion, the mean RDW values change during pregnancy, and some studies have been performed to establish reference intervals in several populations. Recent studies also report various associations between the RDW and pregnancy complications, including anaemia, preeclampsia, diabetes, recurrent miscarriage and others. Further studies are warranted to better evaluate the kinetics of RDW in such conditions, and to establish its potential clinical use.
